# Unparalleled Armour for Aramid Fiber with Excellent UV Resistance in Extreme Environment

**DOI:** 10.1002/advs.202004171

**Published:** 2021-05-06

**Authors:** Fengxiang Chen, Lisha Zhai, Huiyu Yang, Shichao Zhao, Zonglei Wang, Chong Gao, Jingyi Zhou, Xin Liu, Zhenwei Yu, Yong Qin, Weilin Xu

**Affiliations:** ^1^ State Key Laboratory of New Textile Materials and Advanced Processing Technologies Wuhan Textile University Wuhan 430200 P. R. China; ^2^ Beijing Advanced Innovation Center for Biomedical Engineering and Key Laboratory of Bio‐Inspired Smart Interfacial Science and Technology of Ministry of Education School of Chemistry Beihang University Beijing 100191 P. R. China; ^3^ State Key Laboratory of Bio‐Fibers and Eco‐Textiles Qingdao University Qingdao 266071 P. R. China; ^4^ State Key Laboratory of Coal Conversion Institute of Coal Chemistry Chinese Academy of Science Taiyuan 030001 P. R. China

**Keywords:** aramid fibers, extreme environment, mechanical properties, UV resistance

## Abstract

Aramid fibers are widely used in many cutting‐edge fields, including space, aviation, military, and electronics. However, their poor UV resistance and surface inertness seriously hinder their utilization, especially in harsh environments. Here, a dual‐layer ultrathin Al_2_O_3_–TiO_2_ coating with a thickness of 70–180 nm is fabricated on aramid fibers by a modified atomic layer deposition (ALD) method. The tenacity of ALD‐coated aramid fibers decreases only by ≈0.85% after exposure to intense UV light (4260 W m^−2^) under high temperature (>200 ℃) for 90 min, which equals to continuous exposure to sunlight for about 17 500 days. The as‐prepared aramid fibers also show excellent laundering durability, thermal and chemical stabilities. This work presents a green and damage‐free approach to achieve the highly anti‐UV aramid fibers without sacrificing their outstanding performance, which is expected to guide material design for future innovations in functional fibers and devices.

## Graphical abstract

A dual‐layer ultrathin Al_2_O_3_–TiO_2_ coating is fabricated on aramid fibers by a modified atomic layer deposition (ALD) method. The tenacity of ALD‐coated aramid fibers decreases only by ≈0.85% after exposure to intense UV light and high temperature for 90 min. The as‐prepared aramid fibers also show excellent laundering durability, thermal and chemical stabilities.

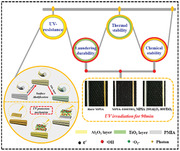

As a high‐performance organic fiber, poly(*m*‐phenyleneisophthalamide) fiber (PMIA, known as aramid fiber 1313) is of great interest for applications in military, aerospace, transportation, electronics, etc., due to their outstanding properties including ultrahigh strength and modulus, lightweight, good fatigue resistance, insulation performance, and flame retardancy.^[^ ^1^, ^2^, ^3^, ^4^ ^]^ Although its performance has significant advantages over ordinary organic polymer fibers, it still suffers from weak heat resistance and poor ultraviolet (UV) light stability compared with many inorganic fiber materials and metal materials.^[^ ^5^, ^6^, ^7^, ^8^, ^9^, ^10^ ^]^ The combination of high temperature and intense ultraviolet radiation can cause a sharp decline in the mechanical properties of PMIA, which largely limits its working durability in the outdoor environment, especially in outer space under extreme ultraviolet radiation and high temperature.^[^ ^11^, ^12^, ^13^ ^]^

A promising strategy to resolve this issue is to coat the polymer fibers with heat‐resistant and radiation‐resistant inorganic materials.^[^ ^14^, ^15^, ^16^ ^]^ Various approaches have been applied to fabricate the inorganic coatings, including in‐site growth, layer‐by‐layer self‐assembly, chemical grafting, sol–gel, and surface coating.^[^ ^17^, ^18^, ^19^, ^20^, ^21^, ^22^, ^23^, ^24^, ^25^, ^26^, ^27^, ^28^, ^29^, ^30^, ^31^, ^32^, ^33^ ^]^ These methods, however, can only tune the thickness of coating in micrometer and/or millimeter scale, which will add significant weight to the microfibers and seriously damage their flexibility. Under UV irradiation, most inorganic materials will generate oxidizing species derived from oxygen‐rich vacancies, nanogranular crystals, and electronical defects,^[^ ^34^, ^35^, ^36^, ^37^ ^]^ which will damage the molecular structure of the PMIA, leading to accelerated aging of the PMIA bulk.^[^ ^28^ ^]^ Thus, it is still a big challenge to develop new coating strategy to achieve high‐performance PMIA fibers suitable for cutting‐edge application in extreme environments.

Atomic layer deposition (ALD) highlights a new direction for functional coating of PMIA, which offers the possibility to construct highly conformal organic/inorganic coatings at lower temperatures with atomic‐level film thicknesses and precise composition control, especially for the sophisticated shape or surface.^[^ ^38^, ^39^, ^40^, ^41^, ^42^, ^43^, ^44^, ^45^, ^46^ ^]^ The tensile property of the spider silk fiber has been enhanced by over 9 times after the controlled ALD of inorganic metal oxides, including Al_2_O_3_, TiO_2_, ZnO for 100–700 cycles.^[^ ^47^ ^]^ However, researches on the assembly of an inorganic coating by the ALD method for improving the integrated function of the aramid fiber such as mechanical properties, thermal properties, radiation resistance, and chemical stability have not been reported. ALD method can only provide the coatings on the fiber surface, whereas aramid fiber has a chemically inert surface that is caused by the strong conjugation effect and steric hindrance effect formed between benzene rings and carbonyl groups in the molecular chain structure. Thus, it is generally difficult to prepare coatings directly on aramid fiber with strong adhesion.

In this communication, we employed a modified ALD method (also known as multiple pulsed infiltration)^[^ ^47^ ^]^ to prepare ultrathin dual coating of Al_2_O_3_–TiO_2_ on PMIA fiber (**Figure** **1** and Figure S1 and Table S1 (Supporting Information)). By infiltrating the precursor molecules into the inside of fiber, they can break the amido bonds in the polymer skeleton resulting in two kind of oxygen‐containing active sites, which will further react with precursors to achieve the successful ALD. In the dual coating of Al_2_O_3_–TiO_2_ for PMIA fiber, the inner amorphous Al_2_O_3_ layer was chosen and utilized as a radical passivation layer and diffusion barrier to effectively block the transition of free radical and avoid the secondary damage of UV‐light‐generated radicals from the TiO_2_ layer, and the outer TiO_2_ layer has excellent UV absorbing capability. The dual layers together protect the fibers from the intense UV‐induced (4260 W m^−2^, Figure S2 (Supporting Information)) and high temperature (>200 ℃, Figure S3 (Supporting Information)) degradation without compromising the desired high performance of fiber bulk. The UV resistances of the ALD‐coated PMIAs in relation to those structures were systematically studied by varying the thickness of Al_2_O_3_ and TiO_2_ coatings. Besides, we also evaluate the laundering durability, thermal and chemical stabilities of ALD‐coated PMIA fibers.

**Figure 1. a,b):**
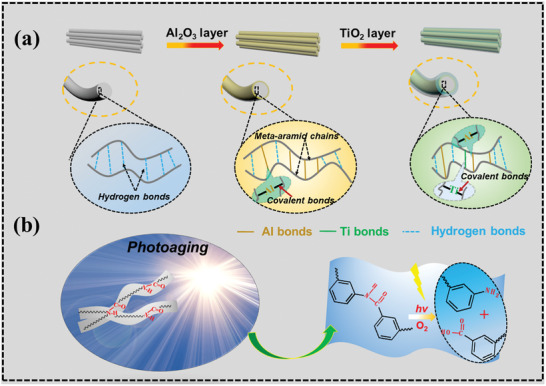
Schematic illustrations for preparing UV‐resistant PMIA by ALD method and the mechanism of improvement of UV resistance of functional nanolayer on PMIA.

Creating the active sites of oxygen‐containing groups on/in the inert PMIA (Figure S4a,b, Supporting Information) is essential for depositing the inorganic and organic materials. The —COOH/—NH_2_ (state I, Figure S4c (Supporting Information)) groups and —COO^−^/—NH_3_^+^ (state II, Figure S4d (Supporting Information)) groups are formed after the trimethylaluminum (TMA) precursor molecules (Figure S4e, Supporting Information) break the —CO—NH— groups on the surface and inside of PMIA organic segment, due to their harsh reaction conditions, including high temperature, high vacuum, and high activity of the precursor molecules. These newly formed active sites can further induce ALD reaction.^[^ ^38^, ^40^, ^41^, ^42^ ^]^ To reveal the possibility of reaction mechanism between precursor Al(CH_3_)_3_ and the PMIA based on what we have observed, spin‐polarized density functional theory (DFT) calculations were utilized. It was found that Al(CH_3_)_3_ molecules can be chemically adsorbed onto either —COOH/—NH_2_ (**Figure** **2**a) or —COO^−^/—NH_3_^+^ groups (Figure 2b) on the surface of PMIA for the formation of —O—Al—(CH_3_)*_n_* with the lower free energy of adsorption of −5.92 and −5.94 eV, respectively. Meanwhile, the Al(CH_3_)_3_ also can be infiltrated into the inside of fiber and adsorbed onto either —COOH/—NH_2_ (Figure 2c) or —COO^−^/—NH_3_^+^ groups (Figure 2d) for formation of —O—Al— with the lower free energy of adsorption of −6.17 and −6.55 eV separately. The driving force for the Al(CH_3_)_3_ adsorption is mainly attributed to the —Al—O bond that is formed by the unsaturated —C=O group and —Al—CH_3_, which is further stabilized by the interface hydrogen bond.^[^ ^45^ ^]^ When the H_2_O precursor is introduced, —O—Al—OH would be produced by hydrolysis reaction with the exposure —CH_3_ group, thereby further repeating the cycles or chemically adsorbing other species and moving forward to the next cycle.

**Figure 2:**
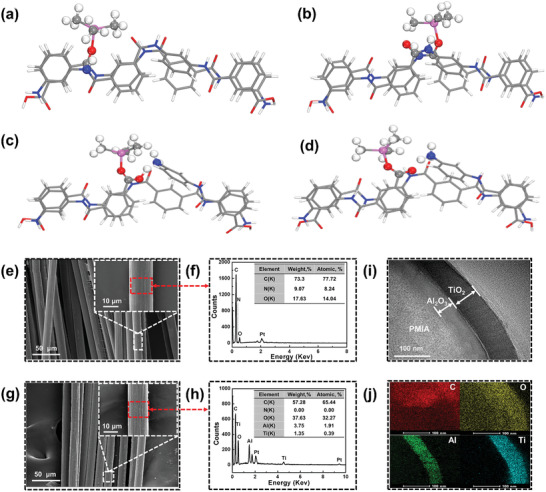
Reaction mechanism of trimethylaluminum (Al(CH_3_)_3_, TMA) on PMIA polymer chain skeleton. Schematic illustrations of Al(CH_3_)_3_ fixed by a) —COOH/—NH_2_ and b) —COO^−^/—NH_3_^+^ through Al—O bonding on the surface of PMIA organic segment and Al(CH_3_)_3_ adsorbed over c) —COOH/—NH_2_ and d) —COO^−^/—NH_3_^+^ through Al—O bonding on the inside of PMIA organic segment. Microscopic topography and composition of the bare PMIA and the ALD‐coated PMIA. e,f) Field emission scanning electron microscopy (FESEM) images of the PMIA at different magnifications and the corresponding EDX curves; g,h) FESEM images of the PMIA–200Al_2_O_3_–800TiO_2_ at different magnifications and the corresponding EDX curves; i,j) TEM image of a cross‐sectional view of the interfacial region and the corresponding mapping of the PMIA–200Al_2_O_3_–800TiO_2_, respectively.

Figure 2e–j shows the morphology and composition of the bare PMIA and the ALD‐coated PMIA. The bare PMIA fibers have a relatively constant diameter of about 15 µm. There are some grooves uniformly distributed on the surface (Figure 2e), which may be caused by the preparation process of the PMIA. PMIA is usually prepared by polycondensation of phenylenediamine and phthaloyl chloride, then pinned into fibers. Energy‐dispersive X‐ray spectra (EDX) show the presence of three elements of C, O, and N, as shown in Figure 2f. ALD‐coated PMIA fibers show a similar morphology with PMIA (Figure 2g). Their strong Ti and Al peaks (Figure 2h) confirm the successful coating of Al_2_O_3_–TiO_2_ layers with excellent conformality of the ALD coatings.^[^ ^48^, ^49^, ^50^, ^51^ ^]^ Transmission electron microscope (TEM) images of a cross‐sectional view (Figure 2i) of the interfacial region was utilized to evaluate the thickness of the ALD coating and the growth rate of different films. For the PMIA–200Al_2_O_3_–800TiO_2_ sample, the thicknesses of Al_2_O_3_ and TiO_2_ coatings are about 37.4 and 58.7 nm, which means that the average growth rates of Al_2_O_3_ and TiO_2_ coatings are 0.18 and 0.07 nm per cycles, respectively. The interfaces between the PMIA and the ALD coating and between the dual ALD coatings appear to be rather smooth, further confirming the excellent conformality feature. Continuous, uniform, and dense Al_2_O_3_ and TiO_2_ coatings on the surface of the PMIA were proved by combining the high‐resolution TEM images (Figure 2i) and EDX mapping (Figure 2j). The above results further approve the theoretical calculation analysis.

X‐ray photoelectron spectroscopy (XPS) was employed to characterize the bonding state of elemental components in bare PMIA, PMIA–1000TiO_2_, PMIA–200Al_2_O_3_–800TiO_2_, and PMIA–1000Al_2_O_3_. As shown in Figure S5a (Supporting Information), the main elements of the bare PMIA were C, O, and N. Characteristic peaks corresponding to the Al and Ti were found on the PMIA–200Al_2_O_3_–800TiO_2_. Two major peaks at 458.5 and 464.5 eV in the narrow spectra of the Ti 2p could be ascribed to Ti 2p^3/2^ and Ti 2p^1/2^, respectively (Figure S5b, Supporting Information). The peak gap (5.7 eV) between the Ti 2p^1/2^ and Ti 2p^3/2^ signals matched well with the Ti in the Ti^4+^ state.^[^ ^52^ ^]^ However, the Al peak was not detected on the surface of PMIA–200Al_2_O_3_–800TiO_2_, which may be caused by the dense TiO_2_ coating. For the PMIA–1000Al_2_O_3_, two symmetric peaks at around 73.8 and 74.4 eV from Al 2p peak could be ascribed to Al—O and Al—OH bonds in the Al_2_O_3_.^[^ ^53^ ^]^ The presence of a large amount of Al—OH on the outermost surface of the Al_2_O_3_ layer provides an activation site for subsequent self‐limiting reaction with ALD TiO_2_. The presence of Ti—O—C bonding in the high‐resolution XPS spectra of C 1s for PMIA–1000TiO_2_ (Figure S5c, Supporting Information) further proved the chemical bonding reaction of ALD at the interface. X‐ray diffraction (XRD) spectrum of the bare PMIA has two strong peaks at 18.5° and 27.18°, corresponding to the (110) and (200) crystal planes, respectively (Figure S5d, Supporting Information). This proves that the PMIA is a semicrystalline polymer.^[^ ^28^ ^]^ The degree of crystallinity is caused by stretching the fibers in the longitudinal direction during the molding process. However, the characteristic crystallization peak of Al_2_O_3_ or TiO_2_ has not been observed in any modified samples, indicating that the coatings deposited by ALD are all amorphous states.

To explore the protecting effect of coatings under UV irradiation, we have prepared a series samples with coating of different thicknesses: PMIA–1000Al_2_O_3_, PMIA–200Al_2_O_3_–800TiO_2_, PMIA–100Al_2_O_3_–900TiO_2_, PMIA–50Al_2_O_3_–950TiO_2_, and PMIA–1000TiO_2_. Their corresponding stress–strain curves before and after UV radiation for 30, 60, and 90 min are shown in **Figure** **3**a–d, respectively. The mechanical parameters are recorded in Table S2 (Supporting Information). The ALD‐coated PMIA exhibited much better mechanical performance than bare PMIA. Before UV irradiation, the tensile strength of PMIA, PMIA–1000Al_2_O_3_, PMIA–200Al_2_O_3_–800TiO_2_, and PMIA–1000TiO_2_ achieved 8.28 ± 0.84, 9.92 ± 0.88, 9.90 ± 0.89, and 10.77 ± 1.05 cN with elongation at break 17.75 ± 1.45%, 19.64 ± 1.62%, 19.14 ± 1.65%, and 22.41 ± 1.68%, respectively. After exposure to UV irradiation (4260 W m^−2^, about 3000 times of the conventional UV light intensity) for 90 min, the tensile strength of the bare PMIA decreased 28.86%, from 8.28 ± 0.84 to 5.89 ± 0.38 cN, indicating poor UV resistance derived from the breaking of amide bonds in molecular chains. However, we noted that the tensile strength of PMIA–200Al_2_O_3_–800TiO_2_ only decreased by 17.07% under 90 min UV irradiation, which is much better than that of the single coating of PMIA–1000Al_2_O_3_ (23.19%) and PMIA–1000TiO_2_ (28.23%). Compared with the original aramid fibers, the tenacity of PMIA–200Al_2_O_3_–800TiO_2_ was only decreased by ≈0.85% after exposure to the UV light (4260 W m^−2^) and high temperature (>200 ℃) for 90 min. Optical images of bare PMIA and PMIA–200Al_2_O_3_–800TiO_2_ under UV irradiation for 30, 60, and 90 min also further approve this analysis. As shown in Figure 3e, bare PMIA fibers rapidly turn yellow under UV light irradiation. With increased irradiation time, the color further deepens until it turns brownish‐yellow. For the PMIA–200Al_2_O_3_–800TiO_2_, it shows only a slight yellowing after UV irradiation for 90 min. These results demonstrate the efficiency of our dual coating approach to achieve highly anti‐UV PMIA.

**Figure 3:**
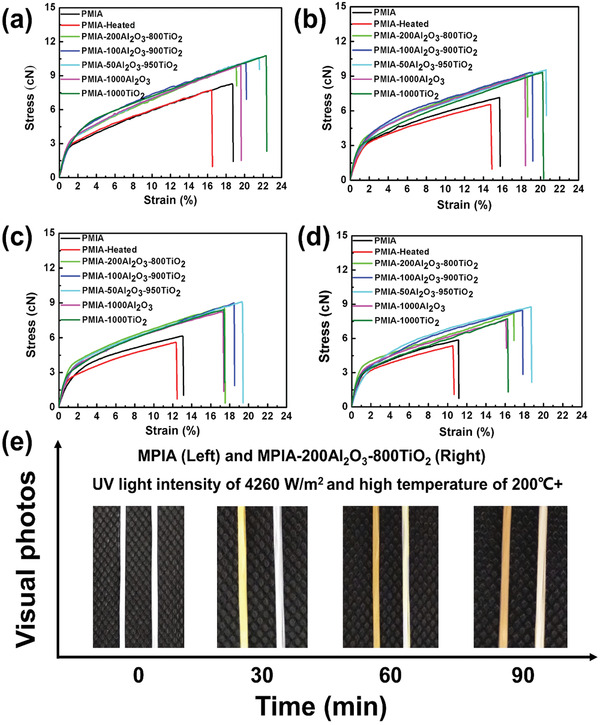
Stress–strain curve of the bare PMIA and ALD‐coated PMIA before and after ultraviolet radiation and the corresponding optical images. a) Stress–strain curve of the bare PMIA and ALD‐coated PMIA before ultraviolet radiation; b–d) stress–strain curves of the bare PMIA and ALD‐coated PMIA after UV irradiation for 30, 60, and 90 min, respectively; e) optical images of bare PMIA and PMIA–200Al_2_O_3_–800TiO_2_ under UV irradiation for 30, 60, and 90 min.

To reveal the effect of ALD coating on the UV resistance of PMIA, we conducted the UV absorption spectra of the bare PMIA and the ALD‐coated PMIA, which are shown in **Figure** **4**a. Both the bare PMIA and the modified PMIA have the same tendency in the range of 200–600 nm. The strength of the ultraviolet absorption peak of the TiO_2_‐coated PMIA increased with the number of sedimentary cycles. By contrast, the UV absorption peak intensity of PMIA–1000Al_2_O_3_ is significantly lower than that of other samples due to the reflection of ultraviolet light by the dense Al_2_O_3_ coating.^[^ ^54^, ^55^ ^]^

**Figure 4:**
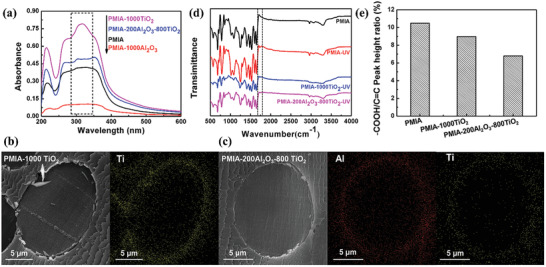
a) Solid ultraviolet absorption spectrum of the bare PMIA and the ALD‐coated PMIA; b,c) SEM images of a ruptured cross‐section sample and the corresponding mapping of bare PMIA and the ALD‐coated PMIA, respectively; d) FTIR spectrum of PMIA before UV radiation, and PMIA, PMIA–1000TiO_2_, and PMIA–200Al_2_O_3_–800TiO_2_ after UV radiation for 90 min. e) —COOH/C=C peak height ratio of PMIA, PMIA–1000TiO_2_, and PMIA–200Al_2_O_3_–800TiO_2_ after UV radiation for 90 min by using normalized treatment based on the characteristic peak of 1610 cm^−1^, respectively.

As we know, TiO_2_ is an excellent semiconductor material with excellent UV response. When the ALD TiO_2_ was exposed to the UV light, the irradiation activated the ALD TiO_2_ to generate strongly oxidative holes (h^+^) in the valence band and reductive electrons (e^−^) in the conduction band.^[^ ^56^ ^]^ These photoinduced electrons are then trapped by the omnipresent molecular oxygen (O_2_) absorbed on the ALD TiO_2_, to yield the anionic superoxide ions (^•^O_2_^−^). Meanwhile, the holes could react with the surface adsorbed —OH/H_2_O by interfacial charge transfer, generating oxygen vacancies (^•^OH). These strong oxidizing species ^•^O_2_^−^ and ^•^OH could move across the sole ALD TiO_2_ layer to reach the surface of the PMIA and cause severe damage to the molecular structure by destructing amide bond and hydrogen bond through a series of irreversible oxidation, degradation, and cross‐linking reactions. Besides, during the ALD process, the modified ALD will also introduce precursor of titanium isopropoxide (TIP) and H_2_O into molecular structures of PMIA,^[^ ^47^ ^]^ which then form TiO_2_ within the molecular chain of PMIA (Figure 4b). This will bring additional structural instability that accelerates the degradation of PMIA under UV irradiation. The introduction of an ultrathin Al_2_O_3_ layer with a thickness less than 37.4 nm could effectively avoid the charge transportation at the interface between the PMIA bulk and ALD TiO_2_ layer, by forming the energies barrier, thereby blocking such active free radicals and electrons to reach the PMIA surface.^[^ ^57^ ^]^ At the same time, this ultrathin Al_2_O_3_ layer can also hinder the infiltration of precursor materials (Figure 4c). Therefore, the UV resistance and antiyellowing performance of PMIA with dual coatings are largely improved, especially in the extreme environment. The characteristic peak —COOH at the Fourier‐transform infrared (FTIR) spectrum (Figure 4d and Figure S6 (Supporting Information)) and —COOH/C=C peak height ratio of PMIA, PMIA–1000TiO_2_, and PMIA–200Al_2_O_3_–800TiO_2_ after UV radiation for 90 min by using normalized treatment based on the characteristic peak of 1610 cm^−1^ (Figure 4e) further convinced our speculation for possible mechanics of photoaging of PMIA fibers (Figure S7, Supporting Information).

Aramid fibers are frequently used for textile, where laundering durability, thermodynamic stability, chemical stability, and flexibility are also crucial. Figure S8a (Supporting Information) shows that the anti‐UV property (indicated by ultraviolet protection factor (UPF) value) of PMIA–200Al_2_O_3_–800TiO_2_ was well maintained and remained at high level (UPF > 70) after 10 accelerated laundering cycles (equivalent to 50 commercial or domestic launderings at 49 ± 2 °C). Thermogravimetric analysis and the corresponding thermogravimetry measurements reveal that the Al_2_O_3_ coating was more effective than TiO_2_ coating in improving the thermal stability of the PMIA, as shown in Figure S8b and Table S3 (Supporting Information). After being immersed in 15% Lithium chloride (LiCl)/Dimethylacetamide (DMAC) solution for 90 min and 85% H_2_SO_4_/65% HNO_3_ mixture for 150 min, respectively, the bare PMIA was completely dissolved. The PMIA–200Al_2_O_3_–800TiO_2_ maintained clear filamentous fibers, showing excellent chemical stability (Figure S8c, Supporting Information). The elastic modulus (Figure S9, Supporting Information), bending length, and rigidity (Figures S10 and S11, Supporting Information) of the PMIA and ALD‐coated PMIAs further proved that ALD coating onto the surface of PMIAs has almost no effect on the elasticity of the PMIA fiber. ALD‐coated PMIAs exhibit similar flexibility to the bare PMIA.

In conclusion, the UV resistance, thermal and chemical stabilities of PMIA fibers have been significantly improved by conformal Al_2_O_3_–TiO_2_ coatings of 70–180 nm thickness prepared using a modified ALD method. The possible ALD reaction route has been proposed based on the DFT calculation. Excellent UV resistance of the as‐prepared aramid fiber was evidenced by showing no yellowing effect after exposure to intense UV light and high temperature for 90 min. The tenacity of the coated aramid fiber only decreased by ∼0.85% compared with the original aramid fiber. Meanwhile, the laminated ultrathin armour on the surface of the PMIA exhibits excellent laundering durability to guarantee a high level of UPF value even after 50 cycles of commercial washing (UPF > 70). The synergistic effect of composite coatings may open a new route for the preparation of functional fibers with superior integrated properties. It will further guide material design for future innovations in functional fibers and devices.

## Experimental Section

##### Chemicals and Materials

Aramid fiber 1313 or PMIA with a diameter of 12 µm was obtained from DuPont Company (USA). In general, the molecular chain of PMIA might have two chemical bond link states, —COOH/—NH_2_ (state I) groups and —COO^−^/—NH_3_^+^ (state II) groups, derived from the breakage of —CO—NH— group, as shown in Figure S4 (Supporting Information). Before ALD, the PMIA was sequentially immersed in absolute ethanol and deionized water and was ultrasonically washed for 60 min. After that, the fibers were dried to obtain clean fibers for further use. Titanium (IV) isopropoxide (99.999%) and TMA (2 m, solution toluene) were purchased from Aladdin Reagent Co., Ltd. Other chemicals, including H_2_SO_4_, HNO_3_, DMAC, LiCl of analytical reagent, were provided by Sinopharm (China), used directly without any further purification. Deionized water was produced by a Milli‐Q Plus 185 water purification system with a resistivity of 10–16 MΩ cm and used in all experiments.

##### Sample Preparation

The Al_2_O_3_ and TiO_2_ layer on PMIA was carried out in a closed‐chamber‐type ALD reactor (D100‐4882, Nuotu, Chongqing) at 150 °C. For Al_2_O_3_ coating, TMA and deionized water were used as precursors. Al_2_O_3_ was deposited according to a typical ALD process in which the cycle consisted of a pulse (TMA)/exposure/purge (N_2_)/pulse (H_2_O)/exposure/purge (N_2_), corresponding to 0.02/12/25/0.1/12/25 s, respectively. ALD TiO_2_ was carried out in similar ways with TIP and deionized water as a Ti precursor and oxygen source. A complete cycle of ALD TiO_2_ proceeded in the following order: pulse (TIP)/exposure (TIP)/purge (N_2_)/pulse (H_2_O)/exposure (H_2_O)/purge (N_2_), with a duration of 0.2/12/25/0.05/12/25 s, respectively. By controlling the cycle numbers, the precise thickness of Al_2_O_3_ and TiO_2_ layers could be achieved. The corresponding samples with various thicknesses of the Al_2_O_3_ and TiO_2_ layers were obtained and the bare and modified PMIA were denoted as PMIA and PMIA–*m*Al_2_O_3_–*n*TiO_2_, where *m*, *n* indicated the ALD cycles for Al_2_O_3_ and TiO_2_, respectively. For comparison, the samples prepared with the same temperature and duration were prepared and marked as PMIA‐heated. Total weight gains of the PMIA after the ALD process were weighted and displayed in Table S1 (Supporting Information).

##### Theoretical Calculation

Spin‐polarized density functional theory calculations were carried out under the scheme of generalized gradient approximation,^[^ ^58^ ^]^ with the use of PBE functional^[^ ^59^ ^]^ and double numerical polarized (DNP) basis, as embedded in DMol3 package.^[^ ^60^, ^61^ ^]^ Global orbital cutoff was applied, with a cutoff radius 3.5 Å. The force on each atom was set as 0.03 eV Å^−1^ for convergence criterion. DNP basis was extensively tested, whose size was comparable to Gaussian basis 6‐311+G** sets, but it offered higher accuracy.^[^ ^62^ ^]^ Among them, the adsorption free energy (AE) was calculated by using the following equation

(1) $$\mathrm{AE}=E\left(X^{*}\right)-E\left(S\right)\;-E\left[\mathrm{Al}{\left(CH_{3}\right)}_{3}\right]$$

where *E*(X*) represents the energy in the adsorption state, *E*(S) represents the PMIA structure energy, and *E*[Al(CH_3_)_3_] represents the Al(CH_3_)_3_ energy, respectively.

##### Physicochemical Characterization

Scanning electron microscopy (SEM, JSM‐6510LV, JEOL Co. Ltd., Japan) was employed to observe the morphologies and microcosmic structures of the samples. The elemental composition of PMIA with and without ALD coating was analyzed using a coupled energy dispersive X‐ray spectroscopy detector (QX200, Bruker). The thickness of the ALD coating on the PMIA surface was characterized by using a TEM (JEOL JEM‐2010) with an acceleration voltage of 200 kV. The PMIA cross‐sectional image was obtained by using a focused ion beam (Helios Lab, operating at 30 kV, USA) technology. The chemical composition of the surface of the PMIA was analyzed by XPS (SPM‐9700, SHIMADZU, Co. Ltd.). The characterizations were conducted at 15 kV using monochromatic Al‐K*α* (1486.6 eV) radiation under ultrahigh vacuum (2 × 10^−9^ mbar). XRD was used to detect the crystal structure of PMIA and ALD PMIA using a D8‐Advance instrument (Bruker). The analysis was conducted by Cu‐K*α* radiation in the 2*θ* range between 10° and 80° at a scan rate of 1° min^−1^.

##### UV‐Resistance Performance

The absorption spectrum of the PMIA with and without the ALD coatings at 200–600 nm was analyzed by an ultraviolet–visible spectrophotometer (Lambda 35, PerkinElmer). Among them, the integrating sphere and BaSO_4_ were used as solid UV test accessories and reference samples, respectively. The UV resistance of PMIA was mainly reflected in the mechanical properties and surface yellowing degree of the fiber. The mechanical property of monofilament of various PMIA was measured with an electronic single fiber tensile strength tester (LLY‐06E, Laizhou Electronic Instrument Co., Ltd.) at 20 °C with a relative humidity of about 63% according to the Chinese standard GB/T14337‐2008. Both ends of the monofilament are fixed by pneumatic clamps with a gauge length of 20 mm and a crosshead speed of 10 cm min^−1^. The measurements for each sample were repeated at least 20 times, and the average value was regarded as the final result. Meanwhile, the optical photographs of PMIA with and without the ALD were taken by a digital camera (Nikon DSLR D5100) under ordinary white light.

##### Chemical Stability Evaluation

0.1 g bare PMIA and PMIA–200Al_2_O_3_–800TiO_2_ were put in 25 mL 85% H_2_SO_4_ and 65% HNO_3_ (wt%: 9:1) mixed solution in a glass bottle. The acid resistance of the samples was evaluated by observing the integrity of the PMIA in the mixed solution after fixed time intervals at room temperature. Photographs were taken with a digital camera (Nikon DSLR D5100) every 10 min until the PMIA was completely dissolved or no longer changed. Also, the solution resistance of PMIA was evaluated using a 15% LiCl/DMAC solution at a temperature of 80 °C. Subsequently, photographs within fixed time intervals were used to analyze the integrity of different samples.

##### Laundering Durability Evaluation

The accelerated laundering durability test of the PMIA was carried out according to the method of AATCC 61‐2009, condition 2A. The stability of the ALD coating was evaluated using a washing laundering machine (model SW‐12A, Changzhou Dahua Electronic Instrument Co., Ltd., China) equipped with a 500 mL (75 mm × 125 mm) stainless steel lever lock canisters. The PMIA (fabrics)–200Al_2_O_3_–800TiO_2_ was cut into 50 mm × 150 mm patches and washed in a rotating closed canister containing 150 mL aqueous solution of an AATCC standard without optical brightener (WOB) detergent (0.15%, w/w) and 50 stainless steel balls, with (2A condition) a water bath at 49 °C, 40 ± 2 rpm. The stability of the UV coating on the surface of the washed PMIA was evaluated by UPF value (one accelerated wash equals 10 commercial or domestic washes).

##### Infrared Spectroscopy Analysis (FTIR)

FTIR spectrometry (Nicolet NEXUS 670, USA) was used to observe the composition of PMIA before and after UV irradiation from 4000 to 400 cm^−1^. An attenuated total reflection mode was adopted for FTIR spectrum.

## Conflict of Interest

The authors declare no conflict of interest.

## Supporting information

Supporting Information

## Data Availability

Research data are not shared.
