# Supplementary material for: Unparalleled Armour for Aramid Fiber with Excellent UV Resistance in Extreme Environment
Source: Adv Sci (Weinh). 2021 May 6;8(12):2004171. doi: 10.1002/advs.202004171 (PMC8224419; doi:10.1002/advs.202004171)
Supplement: Supplementary file 1 — Supporting Information [file ADVS-8-2004171-s001.pdf]

WILEY-VCH

© 2021 Wiley-VCH GmbH

ADVANCED SCIENCE
Open Access

Supporting Information

for *Adv. Sci.*, DOI: 10.1002/advs.202004171

Unparalleled Armor for Aramid Fiber with Excellent UV-Resistance in Extreme Environment

Fengxiang Chen, Lisha Zhai, Huiyu Yang, Shichao Zhao, Zonglei Wang, Chong Gao, Jingyi Zhou, Xin Liu, Zhenwei Yu, Yong Qin*, Weilin Xu**

Supporting Information

Unparalleled Armor for Aramid Fiber with Excellent UV-Resistance in Extreme Environment

Fengxiang Chen, Lisha Zhai, Huiyu Yang, Shichao Zhao, Zonglei Wang, Chong Gao, Jingyi Zhou, Xin Liu, Zhenwei Yu, Yong Qin*, Weilin Xu**

Fengxiang Chen, Lisha Zhai, Huiyu Yang, Zonglei Wang, Chong Gao, Jingyi Zhou, Xin Liu, Prof. Weilin Xu
State Key Laboratory of New Textile Materials and Advanced Processing Technologies, Wuhan Textile University, Wuhan 430200 PR China
E-mail: weilin_xu@wtu.edu.cn

Fengxiang Chen, Zhenwei Yu
Beijing Advanced Innovation Center for Biomedical Engineering and Key Laboratory of Bio-inspired Smart Interfacial Science and Technology of Ministry of Education, School of Chemistry, Beihang University, Beijing 100191, PR China
E-mail: zw_yu@buaa.edu.cn

Fengxiang Chen
State Key Laboratory of Bio-Fibers and Eco-Textiles, Qingdao University, Qingdao 266071, P.R. China.

Shichao Zhao, Prof. Yong Qin
State Key Laboratory of Coal Conversion, Institute of Coal Chemistry, Chinese Academy of Science, Taiyuan 030001, PR China
E-mail: qinyong@sxicc.ac.cn

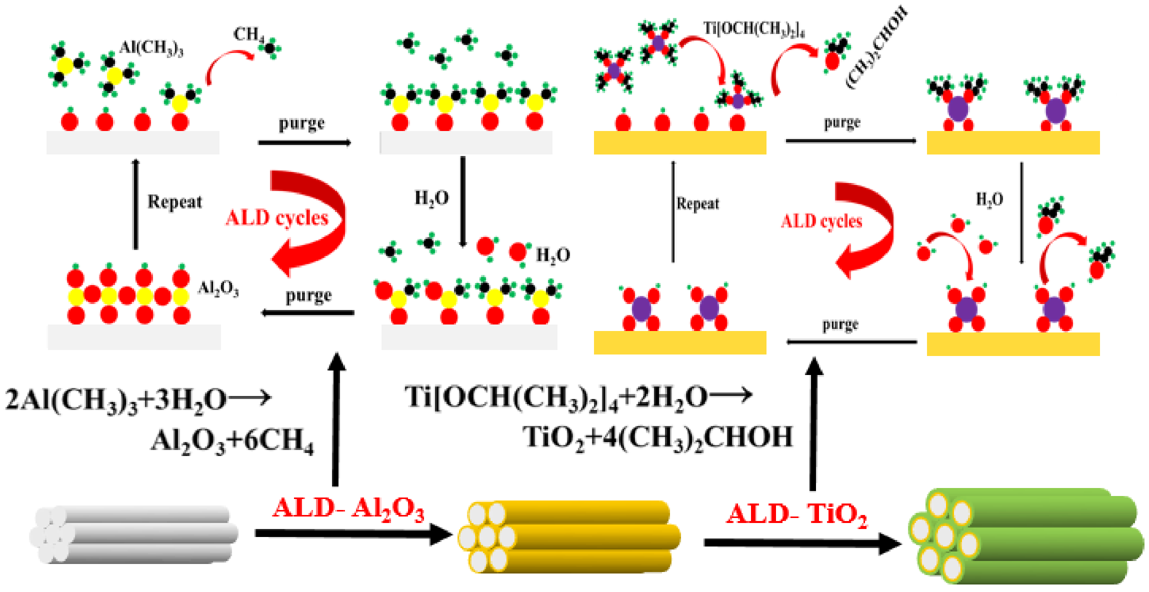

Figure S1. Schematic process of ALD TiO_2 and Al_2O_3 coating on PMIA surface in sequence.

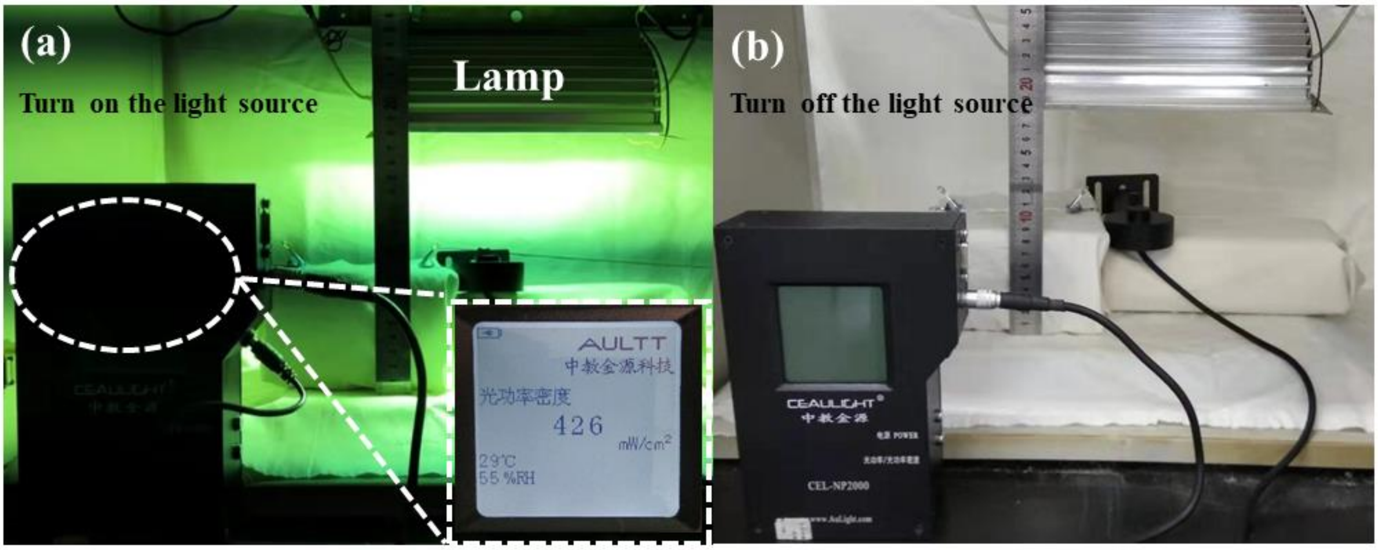

Figure S2. Light intensity during the test.

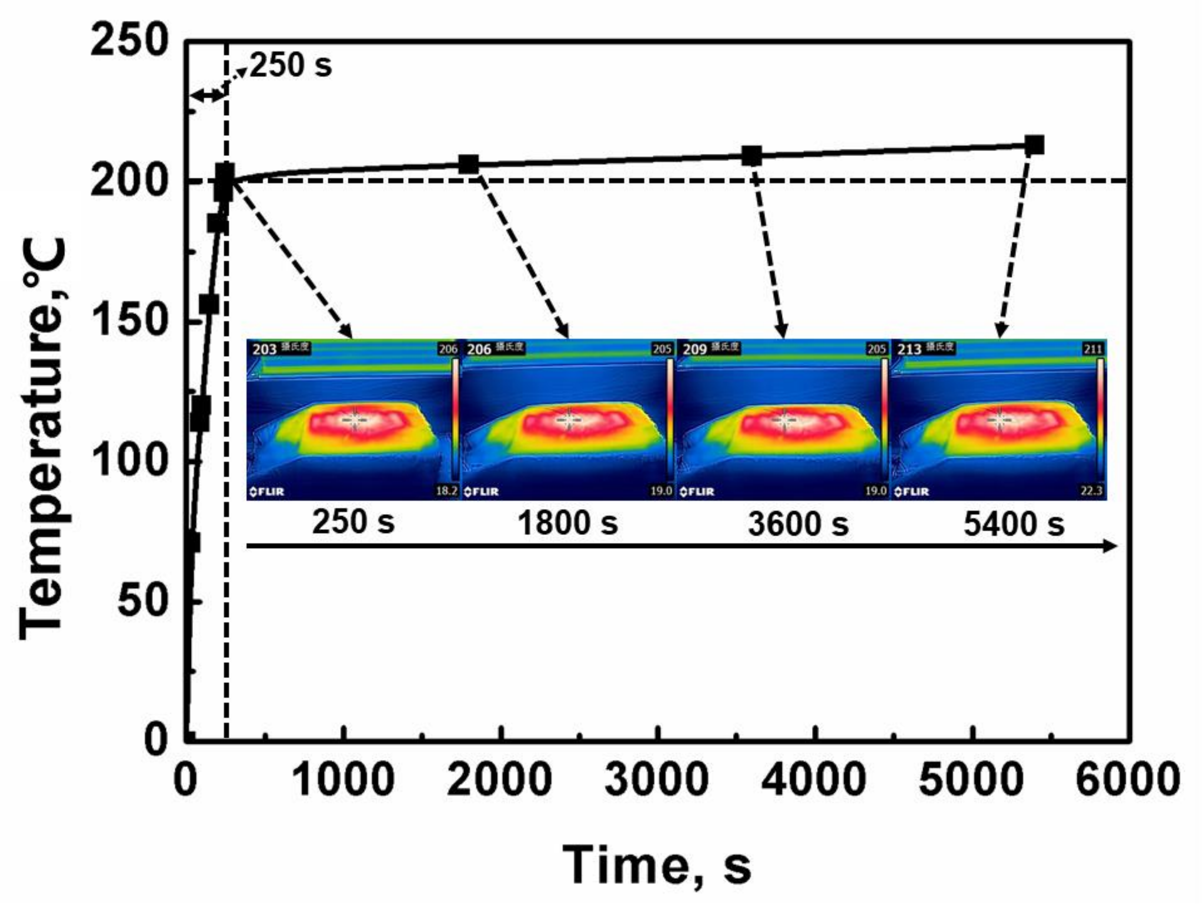

Figure S3. Ambient temperature as a function of the times. The insets are the corresponding thermal image at different times.

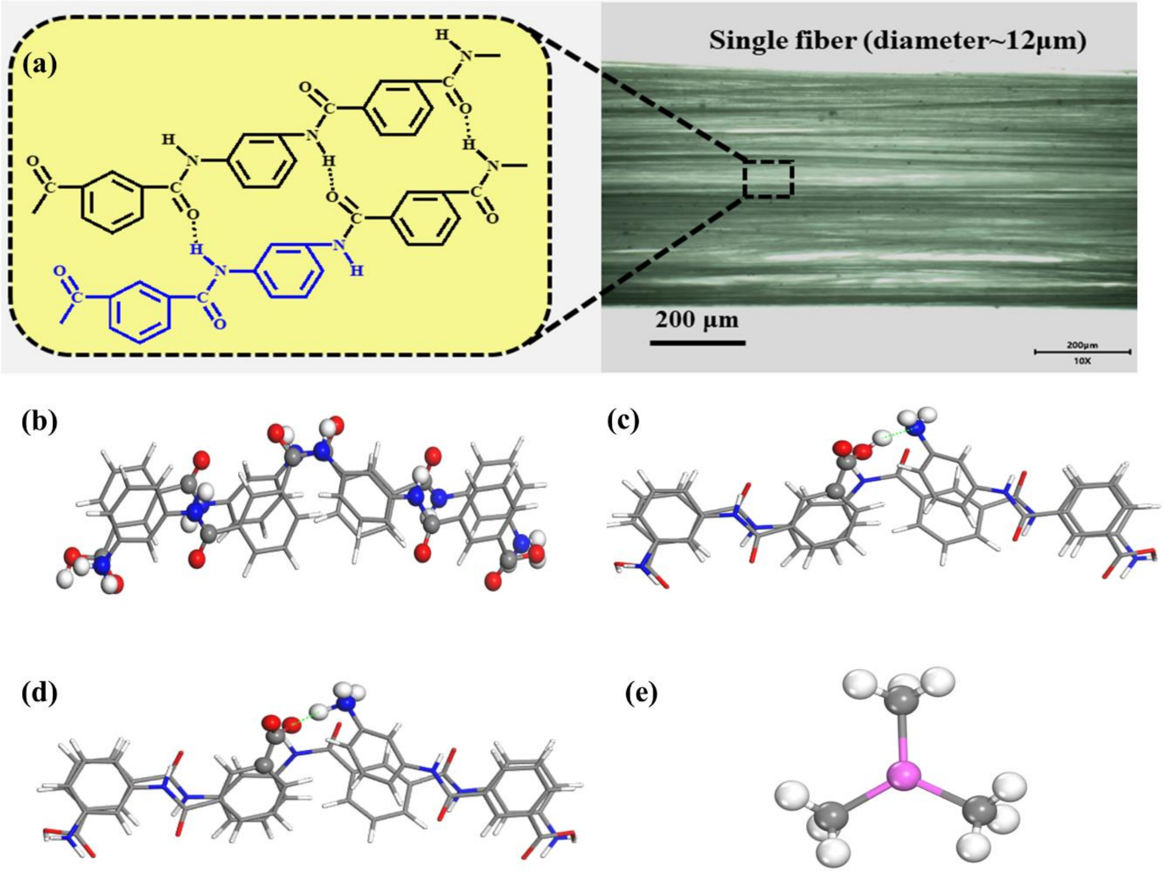

Figure S4. The optic microscope photo, ideal internal molecular structure and computational models of PMIA with the different chemical bond link state. (a) The optic microscope photo and internal molecular structure; Computational models of PMIA (b) with the two chemical bond states, the -COOH/-NH_2 (state I, c) groups and -

COO⁻/-NH_3 ⁺ (state II, d) groups, derived from the contact and infiltration of ALD precursor and then breakage of -CO-NH-group on the surface and inside of PMIA organic segment by a modified ALD and (e) Cluster model of $Al(CH_3)_3$. In these, C, O, N, H and Al are shown as grey, red, blue, white and purple spheres. Benzene fragments in the organic chain are shown as stick for clarity.

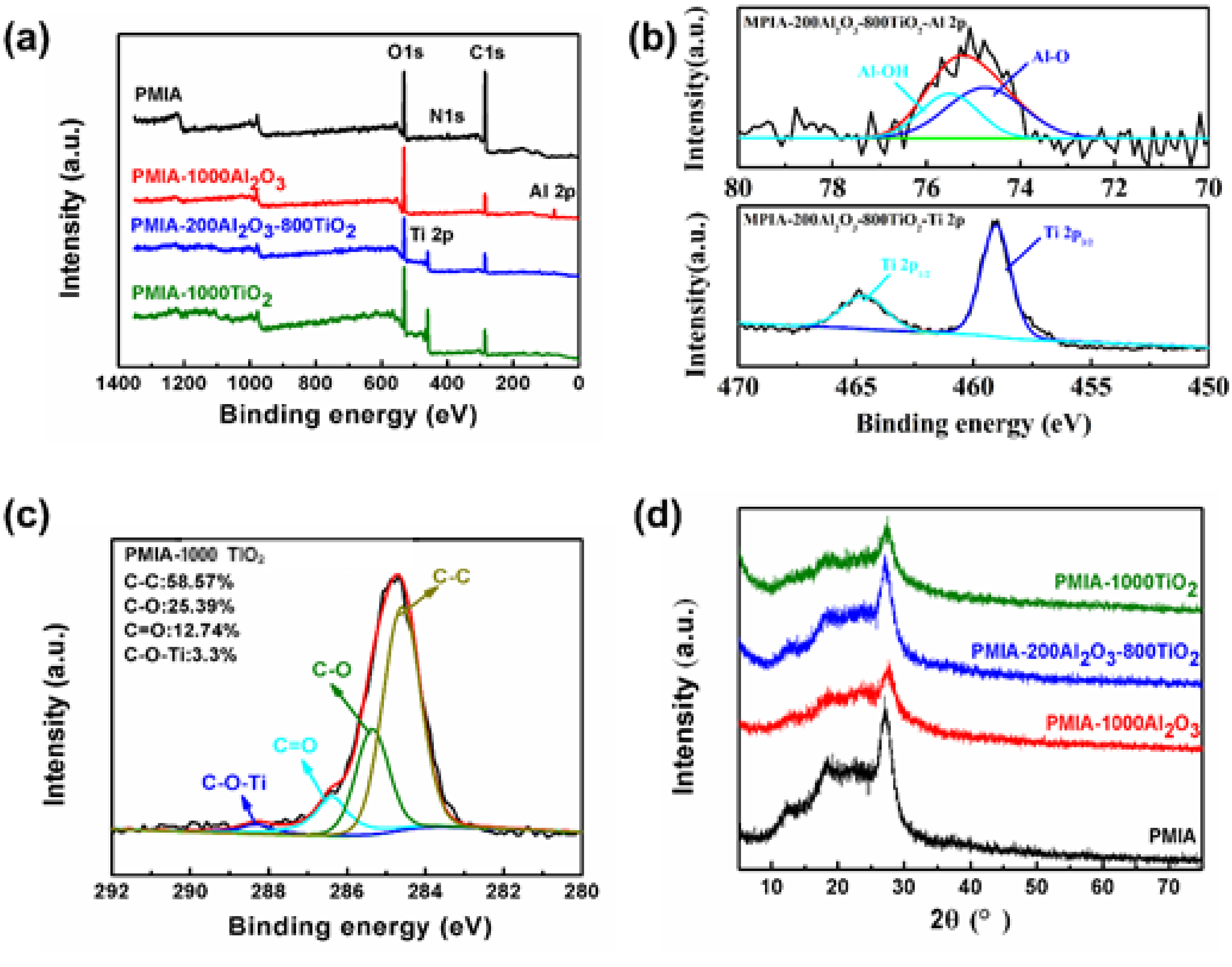

Figure S5. XPS and XRD spectrum bare PMIA and the ALD-coated PMIA. (a) XPS spectrum bare PMIA and the ALD-coated PMIA, (b) high-resolution XPS spectra of C 1s of the bare PMIA and the ALD-coated PMIA, (c) XPS peak spectrum of Al 2p and Ti 2p for PMIA-200Al_2O_3-800TiO_2 and (d) XRD of the bare PMIA and the ALD-coated PMIA.

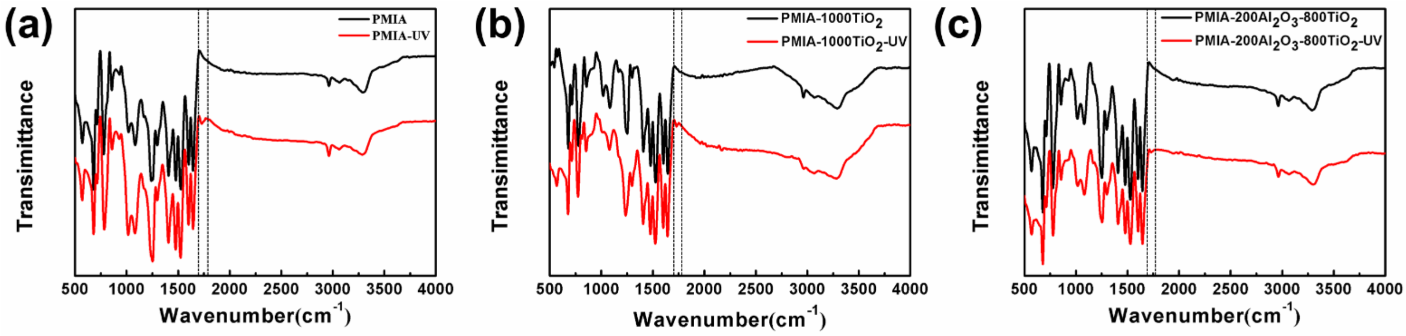

Figure S6. FT-IR spectrum of bare PMIA (a), PMIA-1000TiO_2 (b), and PMIA-200Al_2O_3-800TiO_2 (c) before and after UV radiation for 90 min.

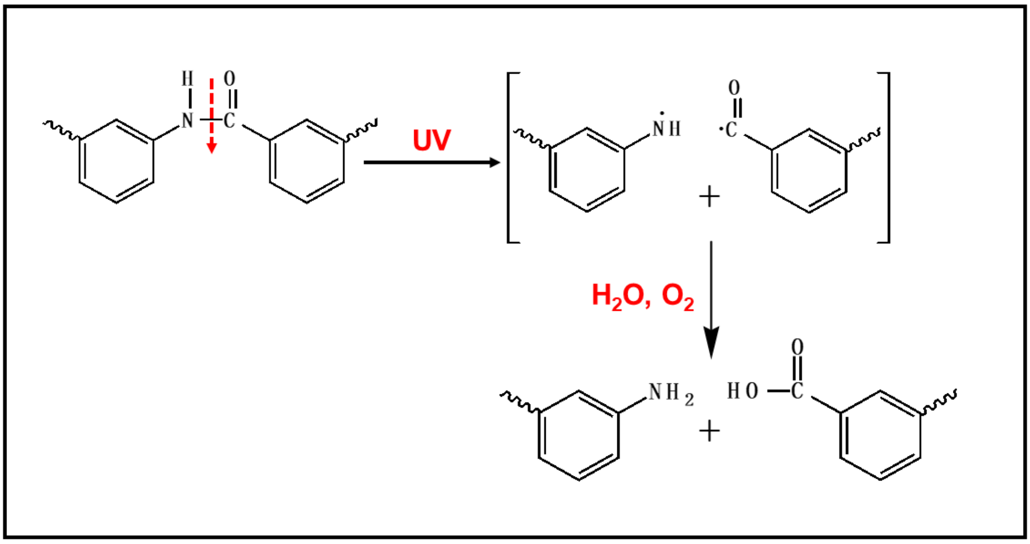

Figure S7. Proposition of the schematic representation of the possible mechanics of photoaging of PMIA fiber.

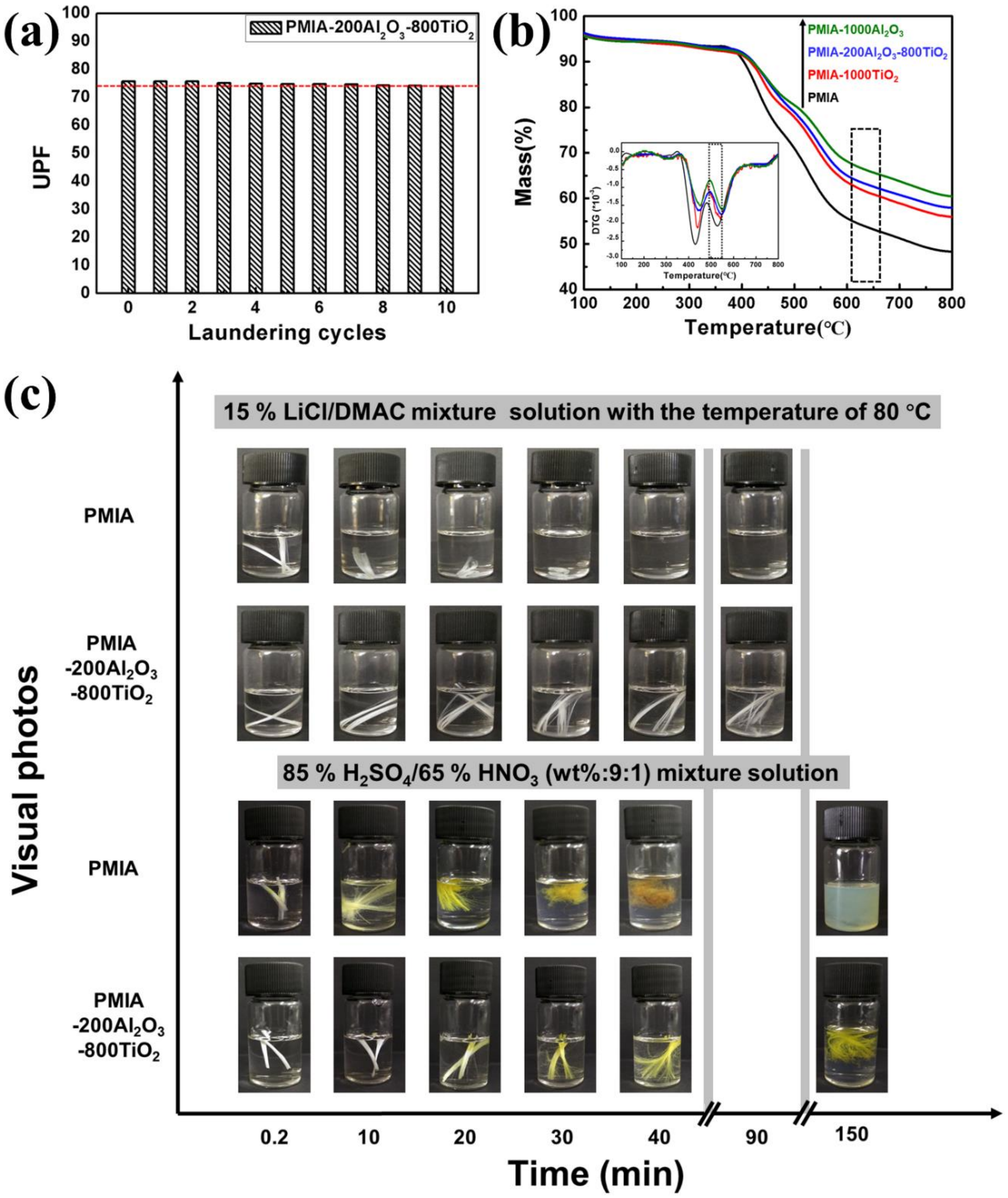

Figure S8. (a) UPF value of PMIA-200Al_2O_3-800TiO_2 after different cycles of laundering durability test; (b) TGA curves for the bare PMIA and the ALD-coated PMIA, the insert is the DTG curves of the bare PMIA and ALD-coated PMIA; (c) Chemical stability of the bare PMIA and PMIA-200Al_2O_3-800TiO_2 in 15 % LiCl/DMAC mixture solution with the temperature of 80 °C (Top) and 85 % H_2SO_4/65 % HNO_3 (wt%:9:1) mixture solution (Bottom).

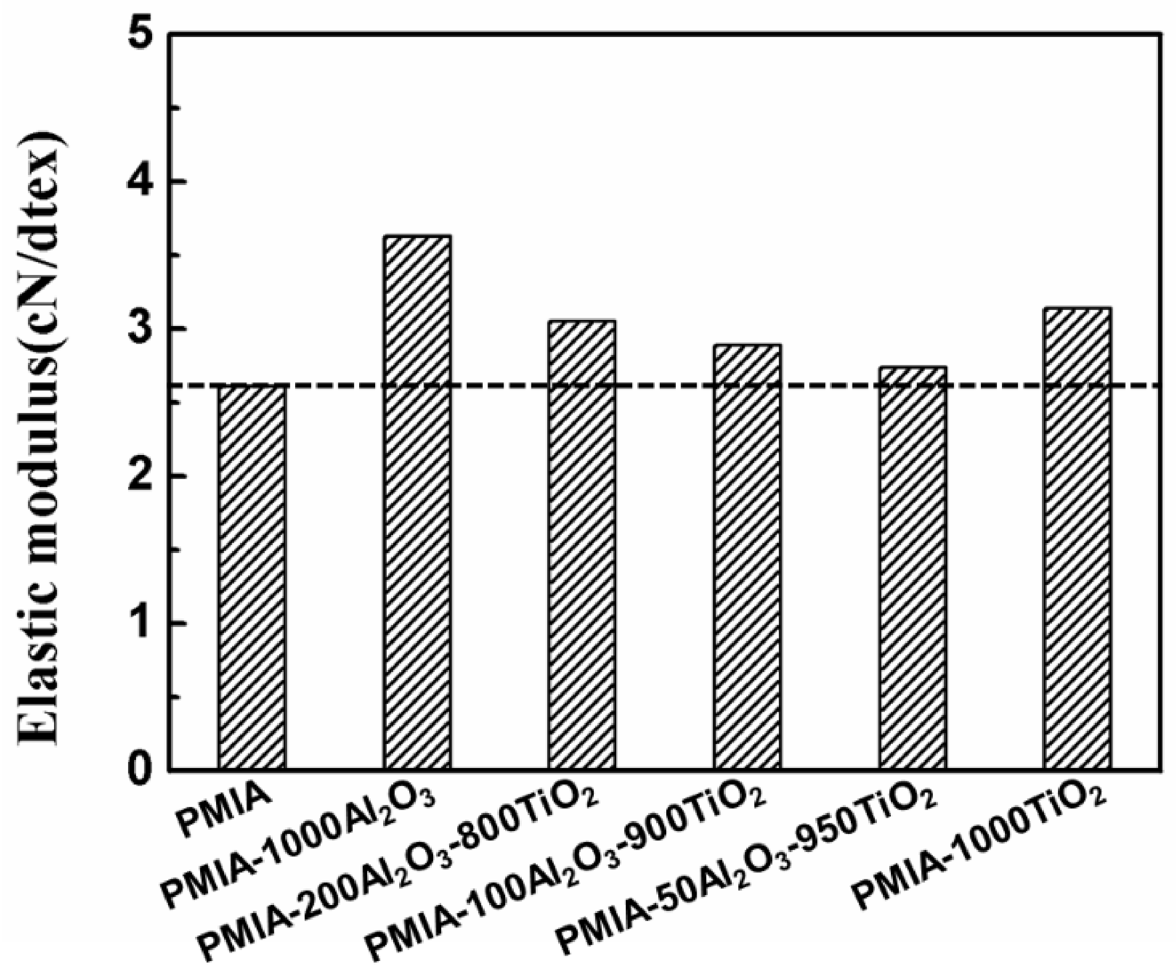

Figure S9. Elastic modulus of PMIA and ALD-coated PMIA.

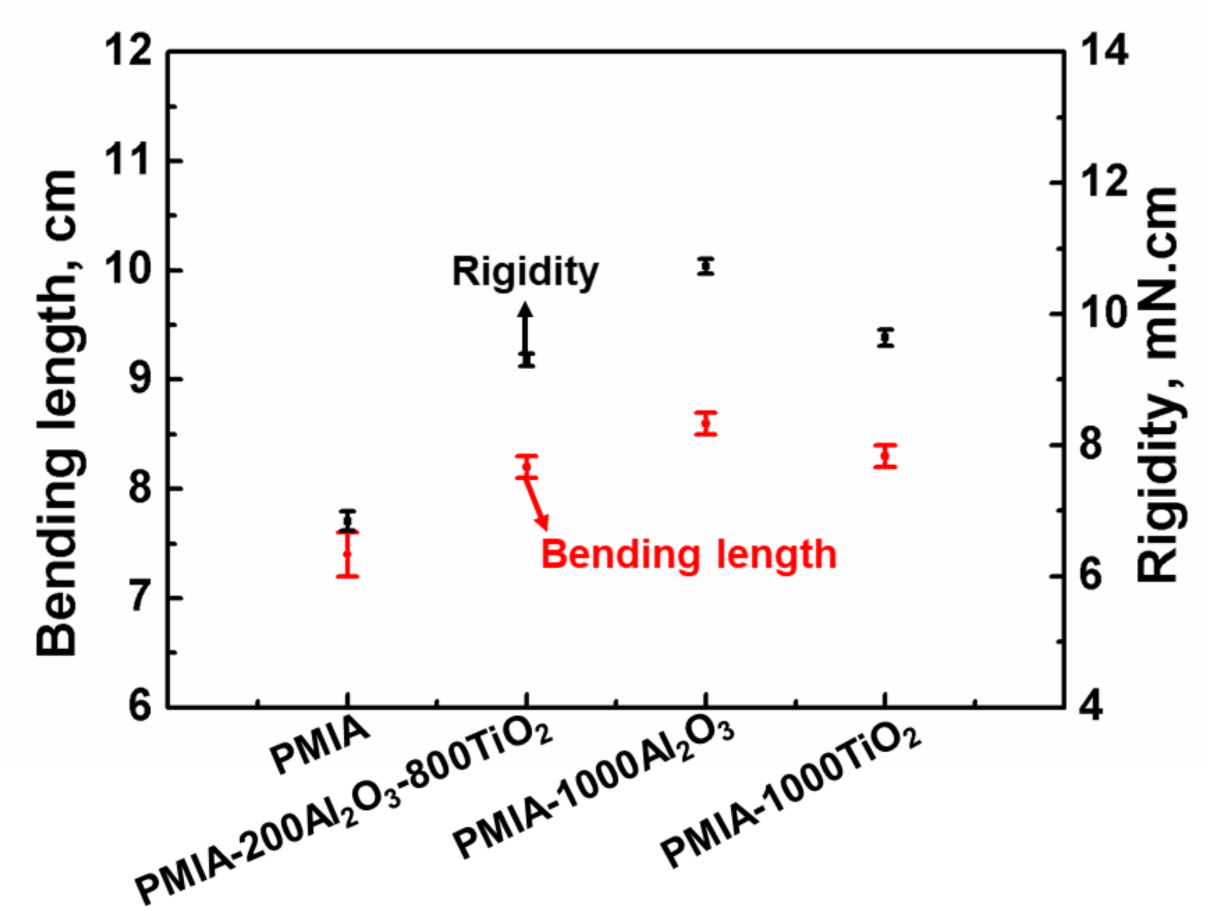

Bending length, cm
Rigidity, mN.cm
12
11
10
9
8
7
6
14
12
10
8
6
4
Rigidity
Bending length
PMIA
PMIA-200Al2O3-800TiO2
PMIA-1000Al2O3
PMIA-1000TiO2

Figure S10. Bending length and rigidity of PMIA and ALD-coated PMIA.

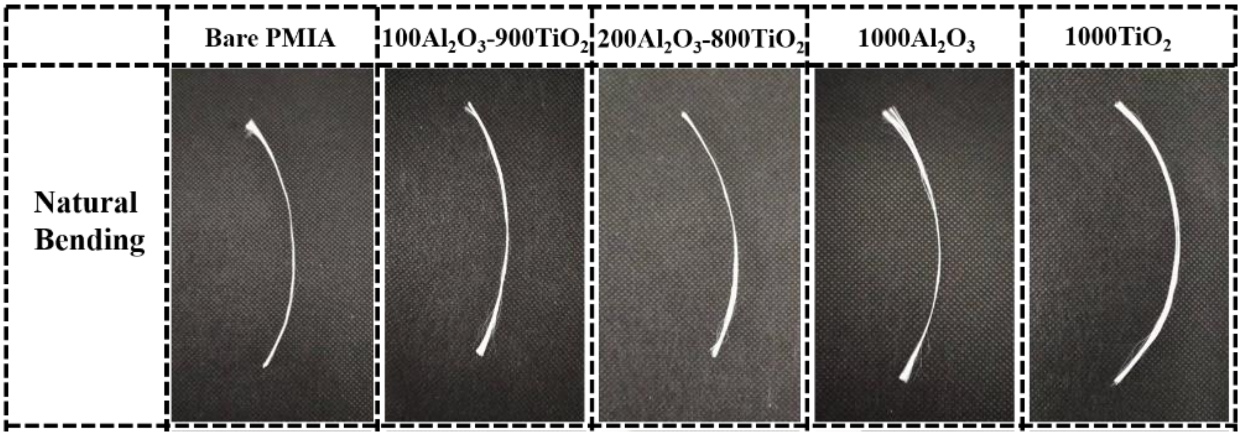

Figure S11. The flexibility of the bare PMIA and the ALD-coated PMIAs.

Table S1

Table S1. Add-on amount of MPIA after ALD treatment.

Sample	Add-on amount (%)
MPIA	/
MPIA-1000Al_2O_3	7.09%
MPIA-200Al_2O_3-800TiO_2	7.96%
MPIA-100Al_2O_3-900TiO_2	8.27%
MPIA-50Al_2O_3-950TiO_2	8.43%
MPIA-1000TiO_2	8.51%

Table S2

Table S2 Mechanical properties of PMIA before and after ultraviolet radiation

Samples	Before UV irradiation		UV irradiation for 30 min		UV irradiation for 60 min		UV irradiation for 90 min	
	Tensile Stress, cN	Strain,%	Tensile Stress, cN	Strain,%	Tensile Stress, cN	Strain,%	Tensile Stress, cN	Strain,%
PMIA	8.28±0.84	17.75±1.45	7.16±0.86	15.75±1.34	6.15±0.52	13.14±1.21	5.89±0.38	11.17±0.97
PMIA-Heated	7.74±0.76	16.52±1.32	6.58±0.68	14.89±1.36	5.62±0.44	12.46±1.24	5.36±0.46	10.68±1.18
PMIA-50Al_2O_3	7.85±0.83	16.53±1.46	7.56±0.72	14±1.23	6.94±0.61	12.65±1.27	6.16±0.42	11.85±1.14
PMIA-100Al_2O_3	8.58±0.90	18.05±1.64	8.06±0.91	16.46±1.45	7.66±0.63	14.54±1.22	7.31±0.53	13.59±1.08
PMIA-200Al_2O_3	9.63±0.87	18.72±1.51	8.94±0.63	17.8±1.48	8.08±0.61	17.16±1.31	7.53±0.57	15.92±1.22
PMIA-1000Al_2O_3	9.92±0.88	19.64±1.62	9.05±0.77	18.41±1.52	8.25±0.72	17.37±1.30	7.62±0.48	16.14±1.17
PMIA-200Al_2O_3-800TiO_2	9.90±0.89	19.14±1.65	9.1±0.92	18.65±1.37	8.72±0.65	17.53±1.31	8.21±0.62	16.93±1.18
PMIA-100Al_2O_3-900TiO_2	10.12±0.94	20.2±1.71	9.32±0.95	19.19±1.51	9±0.84	18.53±1.34	8.49±0.58	17.86±1.25
PMIA-50Al_2O_3-950TiO_2	10.43±0.97	21.61±1.53	9.54±0.73	20.61±1.48	9.1±0.81	19.42±1.25	8.77±0.81	18.77±1.38
PMIA-1000TiO_2	10.77±1.05	22.41±1.68	9.34±0.84	20.32±1.47	8.43±0.72	17.46±1.36	7.73±0.47	16.32±1.29

Table S3

Table S3 TGA and DTG results and the residual weight ratio of PMIA before and after ALD coating

Sample	Peak1	Peak2	Y_c at 800°C (%)
	Temp (℃)	Temp (℃)	
PMIA	429.3	528	48.33
PMIA-1000TiO_2	438.6	540.8	55.95
PMIA-200Al_2O_3-800TiO_2	445.4	546.3	57.94
PMIA-1000Al_2O_3	451.5	551.5	60.45
